# Prediction of genomic breeding values based on pre-selected SNPs using ssGBLUP, WssGBLUP and BayesB for Edwardsiellosis resistance in Japanese flounder

**DOI:** 10.1186/s12711-020-00566-2

**Published:** 2020-08-18

**Authors:** Sheng Lu, Yang Liu, Xijiang Yu, Yangzhen Li, Yingming Yang, Min Wei, Qian Zhou, Jie Wang, Yingping Zhang, Weiwei Zheng, Songlin Chen

**Affiliations:** 1 Wuxi Fisheries College, Nanjing Agricultural University, Wuxi, 214081 China; 2 Yellow Sea Fisheries Research Institute, Chinese Academy of Fishery Sciences (CAFS), Laboratory for Marine Fisheries Science and Food Production Processes, Pilot National Laboratory for Marine Science and Technology (Qingdao), Qingdao, 266071 China; 3 Key Laboratory for Sustainable Development of Marine Fisheries, Ministry of Agriculture, Qingdao, 266071 China; 4 Department of Animal and Aquacultural Sciences, Norwegian University of Life Sciences, PO Box 5003, 1432 Ås, Norway

## Abstract

**Background:**

*Edwardsiella tarda* causes acute symptoms with ascites in Japanese flounder (*Paralichthys olivaceus*) and is a major problem for China’s aquaculture sector. Genomic selection (GS) has been widely adopted in breeding industries because it shortens generation intervals and results in the selection of individuals that have great breeding potential with high accuracy. Based on an artificial challenge test and re-sequenced data of 1099 flounders, the aims of this study were to estimate the genetic parameters of resistance to *E. tarda* in Japanese flounder and to evaluate the accuracy of single-step GBLUP (ssGBLUP), weighted ssGBLUP (WssGBLUP), and BayesB for improving resistance to *E. tarda* by using three subsets of pre-selected single nucleotide polymorphisms (SNPs). In addition, SNPs that are associated with this trait were identified using a single-SNP genome-wide association study (GWAS) and WssGBLUP.

**Results:**

We estimated a heritability of 0.13 ± 0.02 for resistance to *E. tarda* in Japanese flounder. One million SNPs at fixed intervals were selected from 4,978,724 SNPs that passed quality controls. GWAS identified significant SNPs on chromosomes 14 and 24. WssGBLUP revealed that the putative quantitative trait loci on chromosomes 1 and 14 contained SNPs that explained more than 1% of the genetic variance. Three 50 k-SNP subsets were pre-selected based on different criteria. Compared with pedigree-based prediction (ABLUP), the three genomic methods evaluated resulted in at least 7.7% greater accuracy of predictions. The accuracy of these genomic prediction methods was almost unchanged when pre-selected trait-related SNPs were used for prediction.

**Conclusions:**

Resistance to *E. tarda* in Japanese flounder has a low heritability. GWAS and WssGBLUP revealed that the genetic architecture of this trait is polygenic. Genomic prediction of breeding values performed better than ABLUP. It is feasible to implement genomic selection to increase resistance to *E. tarda* in Japanese flounder with 50 k SNPs. Based on the criteria used here, pre-selection of SNPs was not beneficial and other criteria for pre-selection should be considered.

## Background

The Japanese flounder (*Paralichthys olivaceus*) is an economically important aquatic species that is cultured widely in the coastal areas of China, South Korea, and Japan. This fish is popular among consumers for its meat quality and good flavor. In addition, it is also popular among farmers because it is easy to rear, and the costs of flounder farming are moderate. Since the discovery of *Edwardsiella tarda* in Japanese eels in the 1960s, it has become a widely spread pathogen [1]. *E. tarda* causes acute symptoms with ascites in many cultured fish, e.g., Japanese flounder, turbot, and channel catfish [2–5]. Because of the lack of effective measures to control this pathogen, the increasing mortality rate and associated farming costs are now severe problems in the Japanese flounder industry in China. Artificial selection could be a feasible procedure to improve resistance to this disease. Several positive cases of increased resistance to diseases through selective breeding methods have been reported in cultured fish, e.g., Atlantic salmon, rainbow trout, and Atlantic cod [6–14].

In general, resistance to bacterial diseases has a low to moderate heritability in fish, which makes it suitable for family selection [6, 8, 11, 13]. In aquatic breeding, phenotypes are usually evaluated by performing a challenge test, and breeding values of individuals or families are estimated by pedigree-based best linear unbiased prediction (ABLUP). In order to avoid the spread of pathogens from parents to offspring, individuals that fail the challenge test are deemed inappropriate as parents for breeding, but the healthy and unchallenged individuals are selected from families with high estimated breeding values (EBV), which greatly reduces the efficiency of selection for disease resistance.

Genomic prediction (GP) is a powerful tool for estimating breeding values with a higher prediction accuracy than ABLUP [15, 16]. In addition, the use of GP in selection through genomic selection (GS) could solve the problem of selection efficiency in breeding for disease resistance in fish, as the breeding values of selection candidates could be estimated using data from a challenged reference group as soon as the single nucleotide polymorphism (SNP) genotypes are available on the selection candidates. Improvements in sequencing techniques, coupled with their decreasing costs, have enabled whole-genome sequencing in many aquatic species, such as the torafugu (*Fugu rubripes*) [17], Atlantic salmon (*Salmo salar*) [18], Chinese tongue sole (*Cynoglossus semilaevis*) [19], nile tilapia (*Oreochromis niloticus*) [20], large yellow croaker (*Larimichthys crocea*) [21, 22], and Japanese flounder (*P. olivaceus*) [23]. Furthermore, many GP methods have been developed, including BayesB, BayesCπ, genomic BLUP (GBLUP), single-step genomic BLUP (ssGBLUP), weighted single-step genomic BLUP (WssGBLUP), and the stepwise linear regression mixed model (StepLMM) [15, 24–31]. These studies have laid a solid foundation and pave the way for conducting GS in aquatic breeding.

Most reported GP studies in fish have focused on disease resistance [32–38], and some on growth and meat quality traits [39–42]. Liu et al. [32] investigated the feasibility of GP by GBLUP and BayesCπ for *E. tarda* resistance in Japanese flounder through the cross-validation scheme, and revealed the potential of GS based on these two methods for increasing resistance to *E. tarda* in Japanese flounder selective breeding [32]. However, no other genomic method for improving resistance to *E. tarda* in Japanese flounder has been reported, such ssGBLUP, WssGBLUP, and BayesB. Considering the genotyping costs in flounder breeding, it is necessary to evaluate the predictive accuracy of GP methods when marker density is reduced. In addition, we were also interested in the feasibility of GP by using trait-related SNPs. Based on an artificial challenge test and re-sequencing data, the aims of our study were to: (1) investigate the genetic architecture of resistance to *E. tarda* and detect trait-related SNPs; (2) estimate the heritability of resistance to *E. tarda* based on pedigree data; (3) pre-select three subsets of 50 k SNPs from the one million SNPs for GP; and (4) investigate the impact of pre-selection on the accuracy of three GP methods (ssGBLUP, WssGBLUP, and BayesB).

## Methods

### Fish samples and phenotypes

In 2007, three geographical flounder stocks (Korea, Japan, and China) were used as the founder population for developing the 1st generation flounder family [43]. The breeding objectives were to improve disease resistance and growth performance. Every year, we developed 60 to 80 families, with each family cultured in separate tanks under standard culture conditions that were as identical as possible. All fishes were reared and challenged at the Yellow Sea Aquatic Product Company, Ltd., Haiyang, Yantai, Shandong Province, China. The challenge test was started at around 140 days after hatching, using protocols described in Chen et al. [43] and Zheng et al. [44]. In brief, for each family, 100 juveniles were selected at random and challenged with the same concentration of *E. tarda* by intraperitoneal injection. After infection, each batch of 100 juveniles was placed in a separate and sterilized tank with flowing water under culture conditions that were as identical as possible throughout the test. The survival status of the fish was recorded every 6 h. Body length and weight of the fish that died during the test were measured. During the test, the tail fins of dead fish were collected and preserved in absolute ethanol. At the end of the test, tail fins of all surviving fish were also sampled and preserved in absolute ethanol. The experiment ended when mortality stopped. A pre-test was conducted to confirm the concentration of *E. tarda*.

### Genotypes

Genomic DNA was extracted from the tail fin using the traditional phenol–chloroform protocol with RNase treatment. Samples were genotyped by whole-genome re-sequencing on an Illumina HiSeq2000. In total, 1099 flounders were sequenced with an average sequencing depth of 6×. A quality control scheme was performed after re-sequencing and SNP calling. The criteria for removing low-quality SNPs were as follows: (a) a call rate less than 98%; (b) a minor allele frequency (MAF) lower than 5%; and (c) significant deviation from the Hardy–Weinberg equilibrium (*p*-value less than 0.001). All missing genotypes were imputed after quality control. From the remaining SNPs, one million SNPs were extracted at fixed intervals and across the whole genome for downstream studies. Beagle version 3.3.1 [45], PLINK version 1.90b4.4 [46], and fcGENE version 1.0.7 [47] were used for quality control and imputation protocols.

### Estimation of heritability

Heritability for resistance to *E. tarda* in Japanese flounder was estimated by ABLUP with a five-generation pedigree. The binary trait (0 for death and 1 for survival) was analyzed using a threshold model with the probit link function, using R-ASReml [48]. The model was defined as follows:

$$Pr\left( {Y_{ijkl} } \right) = \varPhi \left( {batch_{i} + age_{j} + a_{k} + dam_{l} } \right),$$

where $Y_{ijklt}$ is the observation (dead = 0/alive = 1) for fish $k$ with dam $l$, challenged in experiment batch $i$ at age $j$, $batch_{i}$ is the fixed effect of the experiment batch $i$, $age_{j}$ is the fixed effect of age $j$ (days after hatching) at injection, $a_{k}$ is the random additive genetic effect of fish $k$, $dam_{l}$ is the random maternal effect of dam $l$, $\varPhi$ is the cumulative standard normal distribution function. In this model, the additive genetic effects were assumed to follow a normal distribution $N\left( {0,{\mathbf{A}}\sigma_{a}^{2} } \right)$, where ${\mathbf{A}}$ is the numerator relationship matrix obtained from the pedigree (five generations), while maternal effects were assumed to follow $N\left( {0,{\mathbf{I}}\sigma_{dam}^{2} } \right)$, where ${\mathbf{I}}$ is the identity matrix. Heritability was calculated as:

$$h^{2} = \frac{{\sigma_{a}^{2} }}{{\sigma_{a}^{2} + \sigma_{dam}^{2} + \sigma_{e}^{2} }},$$

where $\sigma_{a}^{2}$ is the additive genetic variance, $\sigma_{dam}^{2}$ is the maternal random effect variance, and $\sigma_{e}^{2}$ is the residual variance and equals $1$.

### Genome-wide association study (GWAS)

To identify SNPs associated with resistance to *E. tarda* in the Japanese flounder, a single-SNP GWAS was performed using PLINK [46] with experiment batch, age at injection, and population structure (the first two dimensional elements of the principal component analysis) as predictors. Genotypes *AA*/*Aa*/*aa* were coded as 0/1/2. The significance threshold was adjusted by the Bonferroni correction as 0.05 divided by the number of SNPs used in the GWAS.

In addition to the single-SNP GWAS procedure, a WssGBLUP was implemented using R-ASReml [48] to identify trait-related SNPs [30]. The model used for WssGBLUP was the same as the model used to estimate heritability, except that ${\mathbf{A}}$ was replaced by ${\mathbf{H}}$, which is the combined genotype- and pedigree-based relationship matrix and its inverse was as follows [28, 29]:

$${\mathbf{H}}^{ - 1} = {\mathbf{A}}^{ - 1} + \left[ {\begin{array}{*{20}c} 0 & 0 \\ 0 & {{\mathbf{G}}^{ - 1} - {\mathbf{A}}_{22}^{ - 1} } \\ \end{array} } \right],$$

where ${\mathbf{A}}$ is the pedigree-based relationship matrix of all challenged animals; ${\mathbf{A}}_{22}$ is the pedigree-derived relationship matrix of the 931 genotyped individuals; ${\mathbf{G}}$ is the weighted genomic relationship matrix constructed by the iterative algorithm described in [30] as follows:

1. set $iter = 0$ and ${\mathbf{D}}_{{\left( {iter} \right)}} = {\mathbf{I}}$, where ${\mathbf{I}}$ is the identity matrix and $iter$ is the iteration number;
2. construct matrix ${\mathbf{G}}_{{\left( {iter} \right)}}^{\varvec{*}}$ as ${\mathbf{G}}_{{\left( {iter} \right)}}^{\varvec{*}}$ = $0.95 *{\mathbf{ZD}}_{{\left( {iter} \right)}} {\mathbf{Z^{\prime}}}\lambda + 0.05*{\mathbf{A}}_{22}$ [26]; $\lambda = \frac{1}{{2\sum p_{j} \left( {1 - p_{j} } \right)}}$, where $p_{j}$ represents the frequency of the second allele at SNP $j$. Matrix ${\mathbf{Z}}$ equals matrix ${\mathbf{M}}$ minus matrix ${\mathbf{P}}$, in which element $M_{ij}$ of ${\mathbf{M}}$ is the genotype of individual $i$ at locus $j$ and element $P_{ij}$ of ${\mathbf{P}}$ is equal to $2p_{j}$;
3. estimate genomic EBV (GEBV, ${\hat{\mathbf{g}}}$) using GBLUP;
4. estimate effects of all SNPs as ${\hat{\mathbf{u}}}_{{\left( {iter} \right)}}$ = $\lambda {\mathbf{D}}_{{\left( {iter} \right)}} {\mathbf{Z}}'{\mathbf{G}}_{{\left( {iter} \right)}}^{* - 1} {\hat{\mathbf{g}}}$;
5. estimate the variance of each SNP $j$ as $\hat{\sigma }_{{u,j\left( {iter} \right)}}^{2}$ = $\hat{u}_{{j\left( {iter} \right)}}^{2} 2p_{j} \left( {1 - p_{j} } \right)$ [49];
6. estimate the element $$j$$ of matrix $${\mathbf{D}}_{{\left( {iter + 1} \right)}}$$ as $$d_{{jj\left( {iter + 1} \right)}}$$$$\frac{{\mathop \sum \nolimits_{j = 1}^{n} \hat{\sigma }_{{u,j\left( {iter} \right)}}^{2} }}{n}$$, where $$n$$ (= 20) is the number of SNPs within a sliding window;
7. normalize matrix ${\mathbf{D}}_{{\left( {iter + 1} \right)}}$ as ${\mathbf{D}}_{{\left( {iter + 1} \right)}} = \varvec{ }\frac{{{\text{tr}}\left( {{\mathbf{D}}_{\left( 0 \right)} } \right)}}{{{\text{tr}}\left( {{\mathbf{D}}_{{\left( {iter + 1} \right)}} } \right)}}{\mathbf{D}}_{{\left( {iter + 1} \right)}}$;
8. construct the matrix ${\mathbf{G}}_{{\left( {iter + 1} \right)}}^{\varvec{*}}$ as ${\mathbf{G}}_{{\left( {iter + 1} \right)}}^{\varvec{*}} = 0.95 *{\mathbf{ZD}}_{{\left( {iter + 1} \right)}} {\mathbf{Z^{\prime}}}\lambda + 0.05*{\mathbf{A}}_{22}$ [26], $iter = iter + 1$;
9. go back to step (4) when $iter$ is less than or equal to 3. Results from the second iteration were used for GWAS analysis. SNPs that explained more than 1% of the genetic variance were considered to be associated with the trait. Manhattan plots were created by R-qqman [50].

### Pre-selection of SNPs

**Fig. 1:**
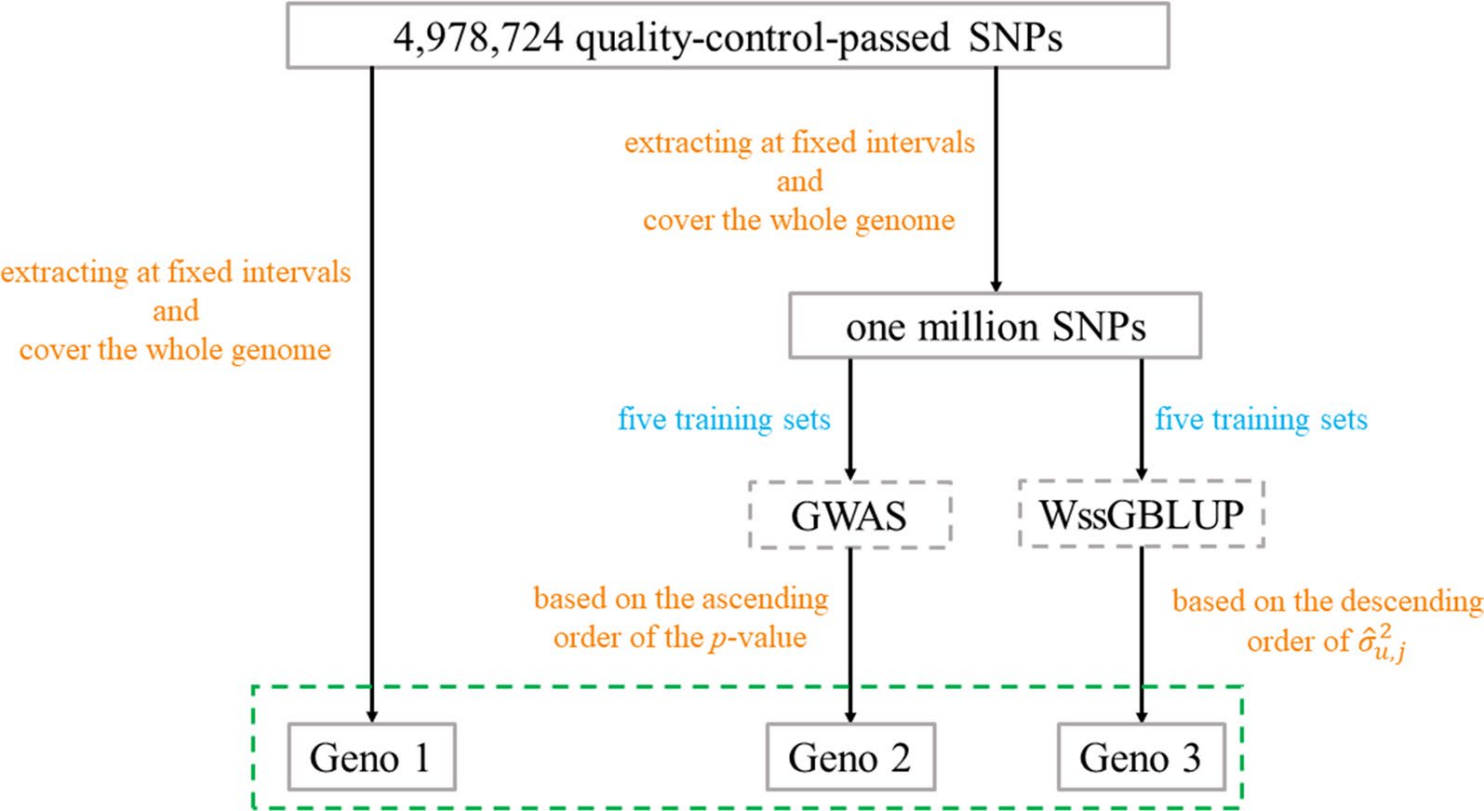
Process of pre-selection of SNPs. Dotted box in gray denotes the analytical method; dotted box in green denotes a pre-selected SNP subset for GS; words in orange denote criteria for pre-selection; words in blue denote the dataset used for pre-selection

To investigate the benefit of pre-selection of SNPs on GP, three genotype subsets were extracted as follows and as illustrated in Fig. 1: (1) Geno1, 50 k SNPs were extracted from all quality-control-passed SNPs at fixed intervals; (2) Geno2, 50 k SNPs were extracted based on the ascending order of the *p*-values obtained by the single-SNP GWAS; (3) Geno3, 50 k SNPs were extracted based on the descending order of the estimate of the variance captured by each SNP ($\hat{\sigma }_{u,j}^{2}$) based on WssGBLUP. To reduce potential biases in accuracy of prediction when Geno2 and Geno3 were used, the *p*-values and the $\hat{\sigma }_{u,j}^{2}$ used for pre-selection were not estimated based on the full dataset, but based on the five training sets that were used in cross-validation. As a result, Geno1 consisted of one subset, while five subsets were generated for Geno2 and Geno3. The distributions of MAF for all quality-control-passed SNPs, and for the Geno1, Geno2, and Geno3 subsets of SNPs were plotted using R-ggplot2 [51].

### Genomic prediction methods and cross-validation

Three GP methods (ssGBLUP, WssGBLUP, and BayesB) were used to estimate GEBV for investigating the potential at improving resistance to *E. tarda* in Japanese flounder breeding programs and to evaluate the effect of pre-selection of SNPs. Pedigree-based EBV (ABLUP) were used as a reference to assess the accuracy of the GP approaches. The model used for GP was the same as that used for the WssGBLUP-GWAS. For ssGBLUP, the ${\mathbf{G}}$ matrix was not weighted, whereas for WssGBLUP, it was weighted based on two iterations. ssGBLUP and WssGBLUP were implemented using R-ASReml [48].

In the BayesB approach, the GEBV ($\hat{g_{i}}$) of individual $i$ is usually defined as $\mathop \sum \limits_{j = 1}^{m} M_{ij} \hat{u}_{j}$, where $\hat{u_{j}}$ is the estimated effect of SNP $j$. In BayesB, a priori, 5% of SNPs were assumed to have non-zero effects, with an inverted chi-square prior [15]. The effects of SNPs in BayesB were estimated by using the MCMC Gibbs sampling scheme with 15,000 iterations and the first 3000 being discarded as burn-in by using R-BGLR [52].

Five-fold cross-validation was conducted to assess the accuracy of the three GP methods. The full dataset of the genotyped individuals was divided into five non-overlapping sets according to their family. One set was used for validation (VDT), in which the phenotypes were masked as missing in the prediction step, and the others were used for training (MDT). The mean area under the receiver operator characteristic (ROC) curves (AUC) of the five VDT, computed using R-pROC [53], was used to evaluate the performance of the GP methods [54, 55]. As mentioned above, each subset of Geno2 and Geno3 was generated based on GWAS and WssGBLUP using each fold of the MDT, and the accuracy of GP with each of the five Geno2 and Geno3 subsets was evaluated based on the corresponding VDT sets.

## Results

### Phenotypes, genotypes and heritabilities

**Fig. 2:**
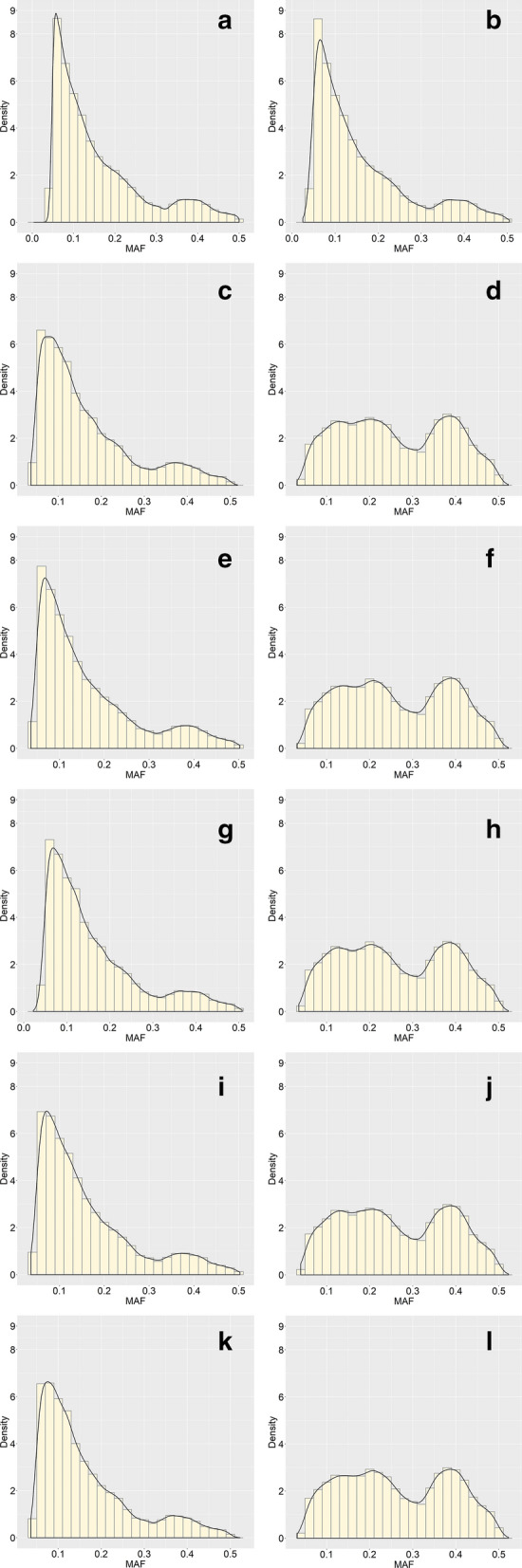
Distribution of minor allele frequency of all (**a**) and pre-selected SNPs based on the Geno1 subset (**b**), the five Geno2 and Geno3 subsets (**c**) to (**l**)

The estimated heritability for resistance to *E. tarda* in Japanese flounder was equal to 0.13, which indicates a lowly heritable trait (Table 1). Based on resequencing data from 1099 flounders, we obtained 9,121,759 initial raw SNPs. After quality control, 4,978,724 were retained for downstream studies. As shown in Fig. 2, the Geno1 subset of SNPs and the full set of SNPs had similar MAF distributions. The five SNP subsets within Geno2 and Geno3 had very similar MAF distributions within the two pre-selection approaches. Compared to the Geno1 subset, the number of SNPs with a MAF between 0.05 and 0.20 was slightly larger in the Geno2 subset. The MAF distribution of SNPs in the Geno3 subset differed from that of the Geno1 and Geno2 subsets, with a smaller number of SNPs with low MAF and a substantially larger number of SNPs with MAF higher than 0.3.

**Table 1:** Estimates (± SE) of variance components and heritability for resistance to *E. tarda* in Japanese flounder estimated by pedigree-based BLUP

$\text{Additive genetic}$	$\text{Dam}$	$\text{Heritability}$
0.16 ± 0.03	0.11 ± 0.03	0.13 ± 0.02

### Genome-wide association study

**Fig. 3:**
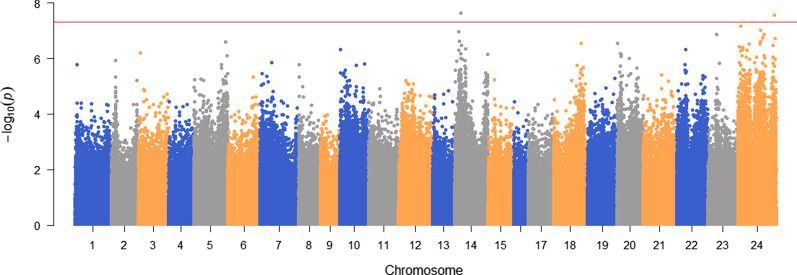
Manhattan plot for resistance to *E. tarda* in Japanese flounder based on single-SNP GWAS

**Fig. 4:**
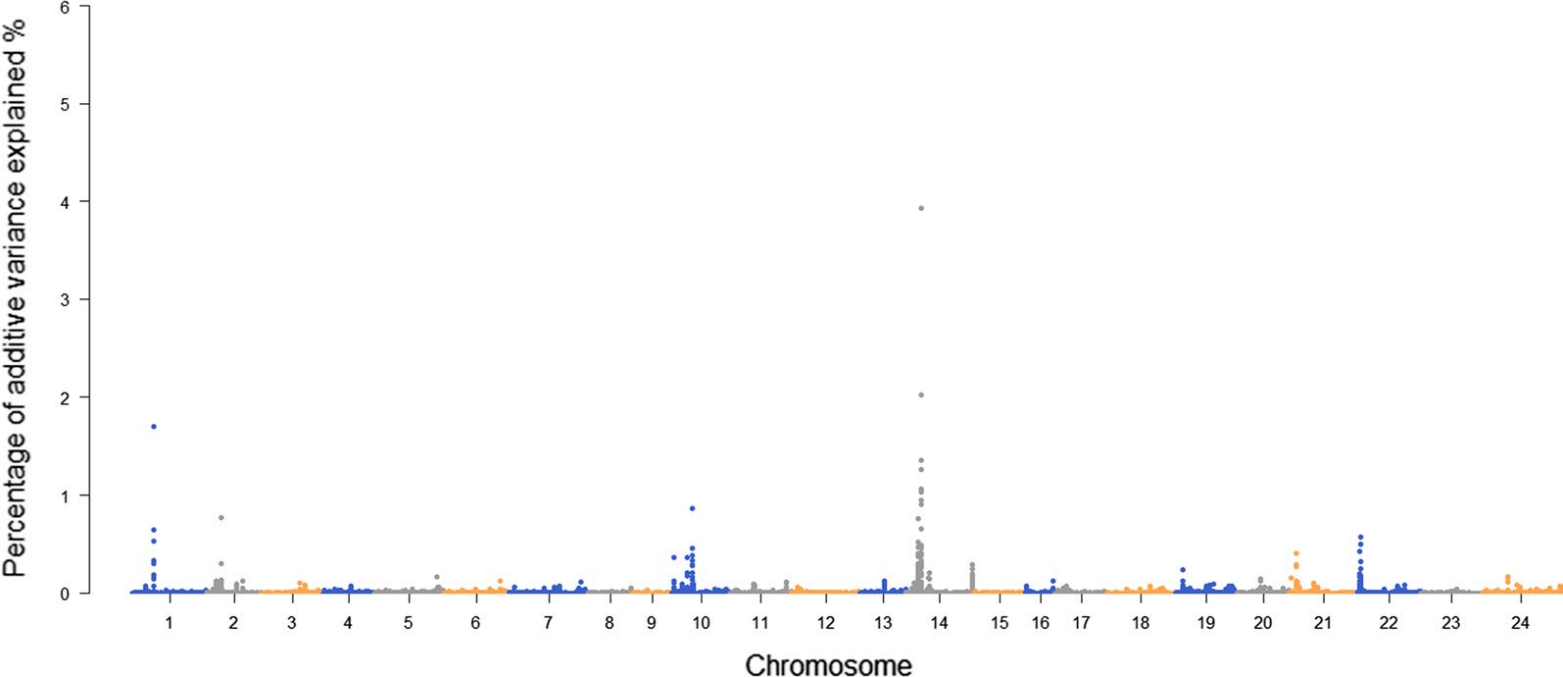
Manhattan plot of the genetic variance explained by each SNP using the WssGBLUP approach

The single-SNP GWAS identified significant SNPs on chromosomes 14 and 24 (Fig. 3). Six SNPs were identified with more than 1% of genetic variance by WssGBLUP, which provides additional support for the significant SNP on chromosome 14 (Fig. 4). Also, one trait-related SNP was detected on chromosome 1 (Fig. 4).

### Genomic prediction and pre-selection of SNPs

**Fig. 5:**
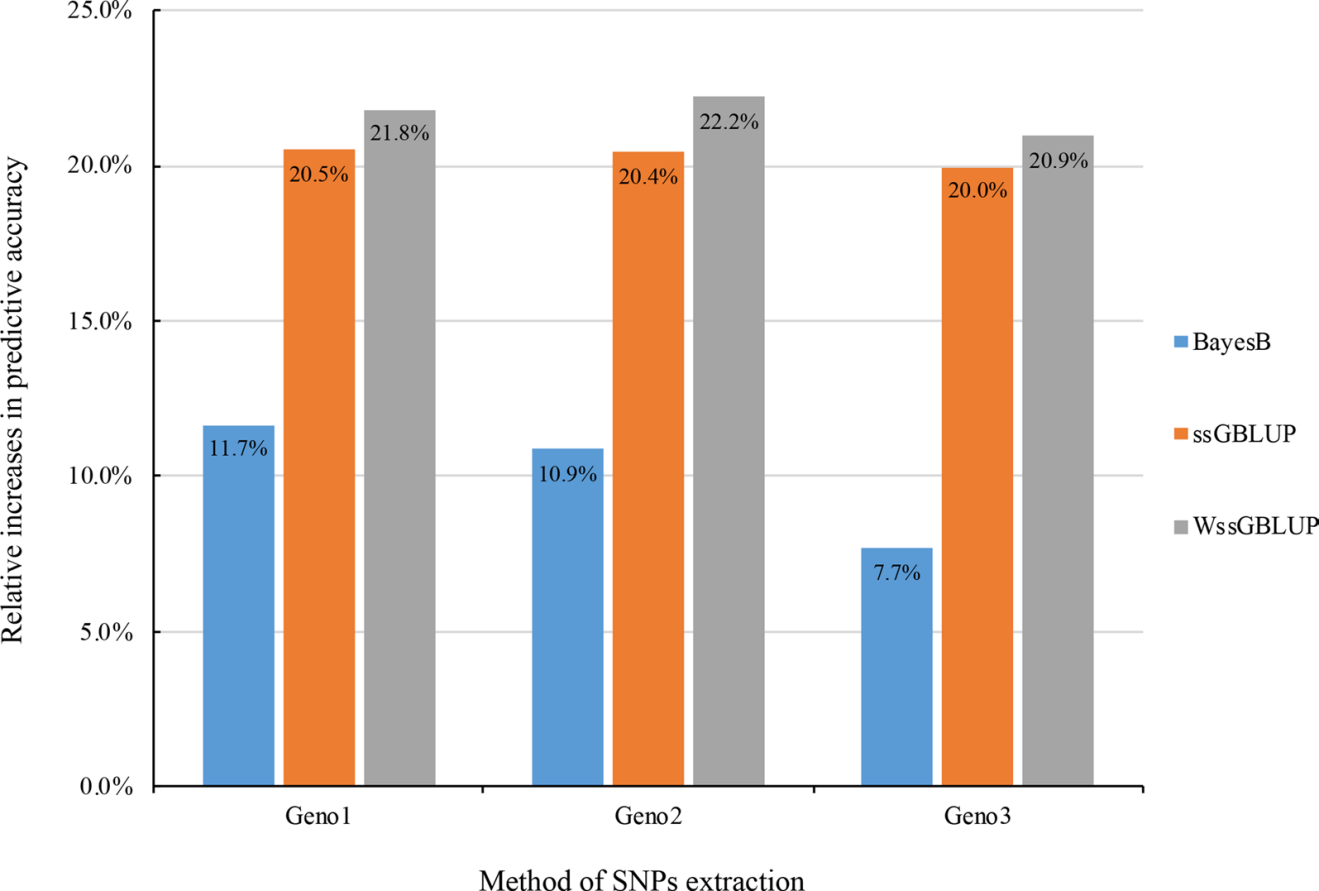
Relative increases of the mean area under the curve of three genomic prediction methods estimated with three subsets of pre-selected SNPs

The accuracy of three GP methods (ssGBLUP, WssGBLUP, and BayesB) with the three subsets of SNPs (Geno1, Geno2, and Geno3) was compared based on their mean AUC in five VDT. The prediction accuracy of all GP methods was superior compared to that of ABLUP, with relative increases of at least 7.7% (Fig. 5). For Geno1, WssGBLUP (0.66) and ssGBLUP (0.65) had close accuracies, which were higher than that of BayesB (0.60). Based on the results of cross-validation, the accuracy of the three GP methods was hardly increased when pre-selected SNPs were used for prediction. Some reductions in accuracy were observed for BayesB and WssGBLUP when Geno3 was used. The mean Pearson’s correlation between EBV and GEBV of VDT ranged from 0.24 to 0.56 with the lowest correlation for WssGBLUP (0.30 in Geno1; 0.24 in Geno2; 0.26 in Geno3), and followed by ssGBLUP (0.37 in Geno1; 0.35 in Geno2 and Geno3). However, GEBV estimated by BayesB had the highest correlation with EBV (0.56 in Geno1; 0.36 in Geno2 and Geno3). GEBV predicted by the three GP methods using the same SNP subset had moderate-to-high correlations (0.67 to 0.94), of which the correlation between ssGBLUP and WssGBLUP (0.94 in Geno1 and Geno3; 0.92 in Geno2) was higher than other combinations (0.67 to 0.87). For BayesB and when using different pre-selected subsets, correlations between GEBV ranged from 0.84 to 0.87, which was lower than observed for ssGBLUP (0.93 to 0.95) and WssGBLUP (0.92 to 0.97).

## Discussion

### Heritability

Disease resistance is a vital trait in aquaculture, particularly in intensive and industrial fish farming, which requires stringent conditions regarding the cultured species and environment. Heritability is an important parameter in selective breeding because it quantifies how much of the phenotypic variance in the trait is caused by genetic factors, which provides a reference for planning breeding schemes. We estimated a heritability of 0.13 (underlying scale) for resistance to *E. tarda* in Japanese flounder, which indicates that it is a low heritability trait. Zheng et al. [44] reported a similar heritability estimate (0.18) based on threshold (logit) model pedigree-based BLUP for resistance to *E. tarda* in Japanese flounder. Studies on another species of flatfish, Chinese tongue sole, estimated heritabilities that ranged from 0.11 to 0.28 for disease resistance defined as a binary trait based on a threshold model [56–58]. Although low, the estimated heritability of 0.13 would allow the flounder breeding industry to improve resistance to *E. tarda* in Japanese flounder by family selection.

### Genome-wide association study

The GWAS detected two significant SNPs on chromosomes 14 and 24. A peak formed around the significant SNPs, which led us to believe that QTL for resistance to *E. tarda* could be located on these chromosomes. WssGBLUP is a powerful tool to detect QTL [30, 59, 60] and the percentage of genetic variance explained by each SNP estimated by WssGBLUP provided additional evidence for QTL on chromosome 14 (Fig. 4). In addition, one SNP on chromosome 1 also explained more than 1% of the genetic variance, thus suggesting the presence of a trait-related SNP on this chromosome. The percentage of genetic variance explained by the single SNPs that we identified here (6 SNPs within a 0.3-Mb window on chromosome 14 with each SNP explaining more than 1% of the genetic variance) was higher than that reported by Palaiokostas et al. (almost 4% of the genetic variance on chromosome 3 in a 0.5-Mb window containing approximately seven SNPs) [33]. This could be due to the different size of the window for weighting SNPs and the method used for estimating the proportion of genetic variance. The performance of WssGBLUP with different weighting methods (single SNP and multiple SNPs as a window) was also investigated to confirm the optimal weighting window (results not shown here). We found that more iterations were needed for stabilization as the size of the window increased. In addition, compared with the weighting of single SNPs, overestimation could be restricted by weighting with an appropriate number of SNPs.

### Pre-selection of SNPs and genomic prediction

There is no doubt that GP methods result in superior prediction compared to pedigree-based BLUP. Our results are consistent with many previous studies in fish [33–38]. Liu et al. [32] reported that GBLUP and BayesCπ based on one million SNPs and 71 candidates resulted in high prediction accuracy in selection, with a Pearson correlation between phenotypes and GEBV of 0.70, which indicates that GS is a potentially efficient method to improve the resistance to *E. tarda* in Japanese flounder. To meet the cost-efficient and time-saving requirements in breeding practice, the predictive accuracy of GBLUP and BayesCπ with a range of SNP densities (1 k, 10 k, 50 k, 100 k, 700 k, and 1 M) was evaluated (results not shown here). We found that GBLUP and BayesCπ yielded similar estimates of GEBV, and that accuracy of prediction was not improved substantially when the number of SNPs was increased beyond 50 k. Therefore, we inferred that 50 k SNPs capture a sufficient amount of information. Based on cross-validation results in this study, we also confirmed that it is feasible to conduct GP with 50 k SNPs to improve the predictive accuracy for resistance to *E. tarda* in Japanese flounder with a relative increase in accuracy of at least 7.7% over pedigree-BLUP. We applied the weighting method that was proposed by Wang et al. [30], which was originally used to improve the power of GWAS for detecting QTL. The weighting method not only improves the precision of GWAS, but also increases the accuracy of GP [59, 60]. In our study, WssGBLUP had the highest relative increases in accuracy over pedigree-BLUP (20.9 to 22.2%), but ssGBLUP resulted in similar accuracies. WssGBLUP is a time-consuming protocol, especially when high-density SNPs are used for prediction. Hence, ssGBLUP might be an optimal method to predict GEBV for resistance to *E. tarda* in our Japanese flounder population.

A previous study on the large yellow croaker (*Larimichthys crocea*) reported that the accuracy of GP for two growth traits could be improved when SNPs were pre-selected based on the largest absolute effects of SNPs and the degree of association with the trait of interest. [40]. In our study, two methods, single-SNP GWAS and WssGBLUP, were used to pre-select 50 k trait-related SNPs, which were then applied in GP. However, no increases in accuracy were observed when the pre-selected SNPs were used, and even some reductions in accuracy were observed for BayesB. This difference in result from [40] could be explained by the dataset used for pre-selection in [40], which was the full dataset instead of the training set. In general, cross-validation is a strategy used for simulating the breeding process of GS to evaluate predictive accuracy. However, to reduce the potential biases caused by SNP pre-selection, the selection of SNPs must be part of the cross-validation approach. I.e. the SNPs must be pre-selected based on an analysis of the training data, and then applied to GP of the validation data.

Because the accuracy evaluated by a cross-validation scheme depends on the relatedness between the MDT and VDT, we also evaluated another grouping strategy to evaluate the feasibility of pre-selection. In this strategy, two individuals were sampled randomly from each family to be used as a VDT, resulting in stronger relationships between the MDT and the VDT. Five replicates of MDT-VDT were generated based on this grouping method. Interestingly, the accuracy based on this grouping method yielded similar results as those reported in Table 2.

**Table 2:** Area under curve from receiver operating curves for resistance to *E. tarda* in Japanese flounder obtained by pedigree-based BLUP and genomic prediction procedures for three methods of pre-selection of SNPs (Geno1, 2, and 3) for five-fold cross-validation

Fold	Pedigree BLUP	Geno1			Geno2			Geno3		
		BayesB	ssGBLUP	WssGBLUP	BayesB	ssGBLUP	WssGBLUP	BayesB	ssGBLUP	WssGBLUP
1st	0.50	0.62	0.63	0.64	0.60	0.64	0.63	0.55	0.63	0.62
2nd	0.54	0.62	0.64	0.64	0.62	0.64	0.64	0.61	0.64	0.64
3rd	0.54	0.57	0.65	0.67	0.52	0.62	0.67	0.54	0.64	0.66
4th	0.57	0.68	0.72	0.74	0.70	0.74	0.73	0.69	0.73	0.72
5th	0.56	0.54	0.62	0.61	0.55	0.62	0.64	0.52	0.60	0.63
Mean	0.54	0.60	0.65	0.66	0.60	0.65	0.66	0.58	0.65	0.65

Pre-selection of SNPs changed the distribution of MAF, with the distribution of MAF in Geno3 being altered more towards high MAF than was obvious for Geno2 (Fig. 2). The proportion of variance explained by a SNP is a function of its allele frequency, i.e. SNPs with a low MAF tend to explain a smaller variance, which could affect the accuracy of GP. However, it is interesting to note that the accuracies of GP methods using Geno2 and Geno3 are very similar, even the Geno3 subset has a higher proportion of high MAF SNPs than Geno2. We speculated that the distribution of SNPs on chromosomes and the linkage disequilibrium between markers and QTL changed when SNPs were pre-selected from the one million SNPs and that their genetic effects and variances had to be estimated again when used for GP. Therefore, SNPs with strong genetic effects or large variances might not explain more variance after re-estimation. Since no clear increase in GP accuracy was observed when trait-related SNPs were used for prediction, pre-selection of SNPs based on variance explained (from WssGBLUP) or *p*-values (from single-SNP GWAS) may not be suitable criteria for pre-selection on the *E. tarda* resistance in Japanese flounder.

## Conclusions

We estimated the heritability of resistance to *Edwardsiella tarda* in Japanese flounder to be 0.13 using a five-generation pedigree. One million SNPs were extracted from the 4,978,724 SNPs that passed the quality control and were used for GWAS analysis and pre-selection of SNPs. Significant SNPs were identified on chromosomes 14 and 24. WssGBLUP with the one million SNPs resulted in the detection of additional trait-related SNPs on chromosomes 1 and 14. It is feasible to implement genomic prediction with 50 k SNPs for increasing resistance to *E. tarda* in Japanese flounder. Pre-selecting 50 k SNPs based on estimates of variance contributed (from WssGBLUP) or *p*-values (from single-SNP GWAS) did not increase accuracy of genomic predictions substantially.
